# Antimicrobial resistance of germs isolated from invasive infections – Romania 2012

**DOI:** 10.1186/1471-2334-13-S1-O16

**Published:** 2013-12-16

**Authors:** Gabriel Adrian Popescu, Roxana Șerban, Ionel Iosif, Irina Codiță, Olga Dorobăț, Daniela Tălăpan, Mariana Buzea, Edit Szekely, Olivia Dorneanu, Karina Bota, Manuela Nica, Raluca Papageorghe, Camelia Ghiță, Irina Nistor, Marina Indrared, Adriana Pistol, Alexandru Rafila

**Affiliations:** 1 Carol Davila University of Medicine and Pharmacy, Bucharest, Romania; 2 National Institute for Infectious Diseases “Prof. Dr. Matei Balş”, Bucharest, Romania; 3 National Institute of Public Health, Bucharest, Romania; 4 Cantacuzino National Institute for Research and Development for Microbiology and Immunology, Bucharest, Romania; 5 Elias University Emergency Hospital, Bucharest, Romania; 6 Tîrgu Mureş Emergency County Clinic Hospital, Romania; 7 University of Medicine and Pharmacy Tîrgu Mureş, Romania; 8 Infectious Diseases Hospital “Sf Parascheva”, Iaşi, Romania; 9 Dr. Victor Babeş Clinical Hospital of Infectious Diseases and Pneumology, Timişoara, Romania; 10 Clinical Hospital of Infectious and Tropical Diseases “Dr. Victor Babeş”, Bucharest, Romania; 11 Colțea Clinical Hospital, Bucharest, Romania; 12 Fundeni Clinical Institute, Bucharest, Romania; 13 Grigore Alexandrescu Clinical Children’s Emergency Hospital, Bucharest, Romania; 14 Bacău County Hospital, Romania

## Background

Antimicrobial resistance has become a serious threat to public health undermining the efficacy of existing antimicrobials (including the last-resort ones) while very few novel antimicrobial agents are in the development pipeline. The interventions aimed to contain antimicrobial resistance need a continuous surveillance of new mechanisms of resistance emergence and the spread of existing ones. Romania participated since 2002 as member of European Antimicrobial Resistance Surveillance Network (EARS) for invasive infection; it is a network which collects data for the most important bacteria and clinically relevant antibiotics. We analyzed the antimicrobial resistance results obtained in 2012 in Romania, in order to support national guidelines for antimicrobial treatment and chemoprophylaxis.

## Methods

Antimicrobial resistance data collected for EARS-Net in 10 public hospitals in Romania in 2012 (756 strains) were analyzed; the resulting levels of resistance were compared with the results from the previous year as presented in EARS-Net 2011 report.

## Results

The number of isolates was 2.55 folds greater than in 2011. Resistance of enterococci to vancomycin is negligible, together with *Enterococcus faecalis* resistance to ampicillin. *S pneumoniae* non-susceptibility to penicillin (38.6%), resistance to macrolides (39.5%) and dual (32.5%) compromise these alternatives for invasive infection treatment; the active options remain: third generation quinolones and vancomycin (both with 100% susceptibility). MRSA represent 53.5% of all isolates; TMP/SMX with 99.1% and vancomycin with 100% susceptibility had greatest activity against *S aureus*. *Escherichia coli* resistance increased for all antibiotics indicated in clinical use (also as MDR – 15.7%) except for carbapenems, and showed a stable increasing trend in last 4 years for quinolones, third generation cephalosporins and aminoglycosides. *Klebsiella pneumoniae* resistant to carbapenems had a sharp increase in the last year (from 0% to 15%) and 42.7% of strains were MDR. Resistance to carbapenems was high for non-fermenters: *Pseudomonas aeruginosa* (61.4%) and *Acinetobacter baumannii* (86.3%); from tested antibiotics, only colistin seems very active in vitro against non-fermenters, but the results were derived from a few number of strains (several laboratories didn’t test for colistin activity).

## Conclusion

The antimicrobial resistance in 2012 reached great levels for many antibiotics. There is an urgent need for a national program and local interventions to stimulate the rational use of antibiotics.

